# Assessing the impact of public funding in alleviating participant reduction and improving the retention rate in methadone maintenance treatment clinics in Taiwan: an interrupted time series analysis

**DOI:** 10.1186/s13012-024-01351-1

**Published:** 2024-02-22

**Authors:** Yu-Chu Ella Chung, Yu-Chi Tung, Sheng-Chang Wang, Chieh-Liang Huang, Lian-Yu Chen, Wei J. Chen

**Affiliations:** 1 Center for Neuropsychiatric Research, National Health Research Institutes, Miaoli, Taiwan; 2 Institute of Health Policy and Management, College of Public Health, National Taiwan University, Taipei, Taiwan; 3 Tsaotun Psychiatric Center, Ministry of Health and Welfare, Nan-Tou County, Taiwan; 4 Department of Mental Health, Ministry of Health and Welfare, Taipei, Taiwan; 5 Institute of Epidemiology and Preventive Medicine, College of Public Health, National Taiwan University, Taipei, Taiwan; 6 Department of Public Health, College of Public Health, National Taiwan University, Taipei, Taiwan; 7 Department of Psychiatry, College of Medicine and National Taiwan University Hospital, National Taiwan University, Taipei, Taiwan

**Keywords:** Methadone maintenance treatment, Public funding, Medical expenditure supplement, Accessibility maintenance, Enrolling, Retention

## Abstract

**Background:**

Given the steady decline in patient numbers at methadone maintenance treatment (MMT) clinics in Taiwan since 2013, the government initiated Patients’ Medical Expenditure Supplements (PMES) in January 2019 and the MMT Clinics Accessibility Maintenance Program (MCAM) in September 2019. This study aims to evaluate the impact of the PMES and MCAM on the enrollment and retention of patients attending MMT clinics and whether there are differential impacts on MMT clinics with different capacities.

**Methods:**

The monthly average number of daily participants and 3-month retention rate from 2013 to 2019 were extracted from MMT databases and subjected to single interrupted time series analysis. Pre-PMES (from February 2013 to December 2018) was contrasted with post-PMES, either from January 2019 to December 2019 for clinics funded solely by the PMES or from January 2019 to August 2019 for clinics with additional MCAM. Pre-MCAM (from January 2019 to August 2019) was contrasted with post-MCAM (from September 2019 to December 2019). Based on the monthly average number of daily patients in 2018, each MMT clinic was categorized as tiny (1–50), small (51–100), medium (101–150), or large (151–700) for subsequent stratification analysis.

**Results:**

In terms of participant numbers after the PMES intervention, a level elevation and slope increase were detected in the clinics at every scale except medium in MMT clinics funded solely by PMES. In MMT clinics with subsequent MCAM, a level elevation was only detected in small-scale clinics, and a slope increase in the participant numbers was detected in tiny- and small-scale clinics. The slope decrease was also detected in medium-scale clinics. In terms of the 3-month retention rate, a post-PMES level elevation was detected at almost every scale of the clinics, and a slope decrease was detected in the overall and tiny-scale clinics for both types of clinics.

**Conclusions:**

Supplementing the cost of a broad treatment repertoire enhances the enrollment of people with heroin use in MMTs. Further funding of human resources is vital for MMT clinics to keep up with the increasing numbers of participants and their retention.

**Supplementary Information:**

The online version contains supplementary material available at 10.1186/s13012-024-01351-1.

Contributions to the literature

- Using interrupted time series regression analysis, we demonstrate that an additional public supplement for the cost of a broad treatment repertoire can boost the enrollment of people with heroin use in methadone maintenance treatment clinics on almost every scale.
- The effect of cost-sharing from public funding is short-term unless sufficient investment is made in the human resources of clinics to keep up with the increasing numbers of participants and their retention.
- Other socioenvironmental factors, such as location and resourcefulness, may impact the efficacy of public funding for methadone maintenance treatment clinics.

## Introduction

The use of illicit drugs has become a major public health issue worldwide, with approximately 275 million people reporting past-year use of any illicit drugs in the World Drug Report 2021 [1]. Specifically, the number of people who had past-year use of opioids reached more than 61 million, or 1.22% of the global population, in the 2022 report [2]. Since opioids account for two-thirds of drug-related deaths, mostly from overdoses, the control of their use and the coverage of related treatments have become challenging global issues [2].

Harm reduction programs are effective measures for decreasing the harm caused by the use of opioids, particularly by injection, mainly via medication-assisted treatment using methadone [3]. However, despite the widespread adoption of harm reduction programs among Western countries after the mid-twentieth century, similar programs were not initiated in Asian countries until the last decade of the century [4]. The slow adoption in Asia might result from concern about the spillover effect of harm reduction programs, such as conflict with past mainstream government approaches that viewed addiction as a “crime” rather than a “disease” [5], anxiety about the potential diversion of opioid agonist medications [6], and the debate about whether applying substitute drugs as treatment would send the wrong message to the public [7].

Not until 2006 did the Taiwanese government initiate its own harm reduction program to mitigate the rapid growth of the human immunodeficiency virus (HIV) epidemic among people with injection drug use [8]. The nationwide harm reduction program consisted of three-pronged policies, including the expansion of extant education and screening, a needle-syringe program (NSP), and opioid substitution therapy (OST) [9]. At the beginning of the OST, only methadone maintenance treatment (MMT) was provided until the option of buprenorphine maintenance treatment became available nationwide in 2010. Although Taiwan implemented national health insurance (NHI) in 1995 to provide general health services, the expenditure for treatment for drug dependence is explicitly excluded from this coverage by law. Thus, the government allocated a special budget to decrease patients’ copayment for the cost of MMT. The number of participants receiving MMT grew rapidly in the first 3 years to approximately 12,590 participants per month in 2008 [8], but it gradually dropped to 8,000 participants or less per month in 2017 [10]. Nevertheless, people with heroin use who attended MMT clinics were found to have a better quality of life than those who did not attend MMT clinics [11], and participants with a longer cumulative MMT duration were associated with lower all-cause and drug-related mortality rates [12].

To enhance the overall capacity of treatment for people with illicit drug use disorders, the government in 2017 adopted the “New-Generation Strategy to Combat Drug Abuse” (hereafter referred to as the new-generation strategy), in which governmental sectors across law enforcement, education, and health and welfare were incorporated in a united task force. With support from this strategy, the Ministry of Health and Welfare (MOHW) successively launched two funding programs to alleviate the decline in MMT participants: the Patients’ Medical Expenditure Supplements (PMES) program in January 2019 and the MMT Clinics Accessibility Maintenance (MCAM) program in September 2019. The issue now is how to evaluate the impact of these two programs in an appropriate policy implementation framework.

### Policy implementation framework

Based on the two-part conceptual framework of implementation synthesized in a recent review [13], the implementation of these two funding programs on MMT clinics in Taiwan could be described in two parts: (1) the process model of implementation consisting of policy package and process, and (2) determinants framework consisting of policy instruments, strategies, and policy context.

### Process model of implementation—policy package and process

The opening of MMT clinics in any medical institution requires the signing of contracts with the MOHW. Based on resourcefulness in service, the MMT clinics were categorized into three facility levels: core hospitals, hospitals, and clinics. All of the treatment plans, treatment procedures, space planning, and storage plans for controlled drugs in MMT clinics must be inspected regularly by the local government. The set-up of MMT clinics was first as a pilot in four major sites in 2005 and then expanded to every city and county in 2006 [8]. After a dramatic decline in the incidence of HIV infection among people with injection drug use in 2007 [14] owing to a fast implementation that was rated as a successful model in a systemic review [15], the MMT program continues to be an important component of the harm reduction for people with heroin use in Taiwan.

Patients attending the clinic will receive a full subsidy for the test fee for infectious diseases and for the methadone medication fee, as well as a partial subsidy for the remaining tests by the MOHW. If a patient is diagnosed with HIV infection, all the aforementioned costs from the MMT become fully subsidized. When legal amendments to allow deferred prosecution nationwide were enacted in 2008, the number of participants receiving MMT peaked in this year [8]. Facing the challenge of the gradual decline in the number of MMT participants afterward, the new-generation strategy provided the MOHW extra resources to successively launch the PMES and MCAM.

### Determinants framework

#### Policy instruments and strategies

The legislative changes for setting up the “Drug Use Prevention Fund” for the new-generation strategy have served as the major financial system infrastructure in the implementation of MMT policy interventions. In 2017, the central government amended “Narcotics Hazard Prevention Act” to set up an independent fund to support programs related to drug abuse prevention and treatment in related governmental sectors. The sources of funding include regular government budget, fines due to the violation of the law, donations, and any other possible income related to drug use regulations. The total funding amount in 2019 was NT$361.10 million (US$11.67 million). Hence, the set-up of “Drug Use Prevention fund” has secured the maintenance of drug-related policy interventions.

Other strategies included building up a “National Case Management System of Drug and Alcohol Use” and connecting to the existing medical information system used by every MMT clinic. The applications for subsidy are conducted online to minimize the administrative burden of the staff in the MMT clinics. Additionally, advertisements and health education leaflets are sent to each MMT clinic to enhance the dissemination. Another strategy was to maintain constant communications between the central government and local governments to assist MMT clinics in participating in these policy interventions. Lastly, the MMT clinic's participation rate in the policy interventions was chosen as an indicator for the performance evaluation of each local health agency.

The close connections developed over decades between the MOHW and medical institutions nationwide might also be beneficial to the implementation process. In particular, the connections have been strengthened since the launch of NHI, which has covered approximately 99.99% of the citizens and contracted 92.04% of the medical facilities (e.g., hospitals, clinics, and pharmacies) [16].

##### Context of the MMT policy intervention

Starting in January 2019, the PMES program was launched to further subsidize necessary procedures for people with heroin use attending MMT clinics, including additional assessments, psychotherapy, and miscellaneous expenditures (see more details in Supplementary Table S1). Nevertheless, patients typically need to pay the remaining costs since the PMES sets a yearly cap for each patient. With an average median household income of $NT 905 thousand (US$ 29.26 thousand) in 2019 [17], the total funding amount of the PMES in the same year was approximately $NT 98.36 million (US$ 3.18 million). Almost 100% of the subsidization applications for the items in the PMES were approved. Among the total amount subsidized, the top 5 categories were fee for assessment at outpatient clinics (30.02%), urine drug tests (18.78%), case management (11.21%), individual psychotherapy (10.61%), and diagnostic interview (7.14%). In specific items, the fee related to psychotherapy, including individual and group psychotherapy, accounted for 15.49%.

As the number of patients per MMT clinic continued to dwindle during the implementation of the PMES program, the MCAM program was launched in September 2019 to help medical institutions with MMT clinics serving a monthly average number of daily participants of 150 or less by their capacity levels: tiny (1–50), small (51–100), and medium (101–150). For MMT clinics with larger capacities that were not eligible for the MCAM, we designated their scale as “large”, i.e., monthly average number of daily participants of 151 to 700. To compensate for the cost of MMT clinics with smaller capacities, the ceilings of yearly MCAM funding are set at $NT550 thousand (US$17.78 thousand) for tiny clinics, 350 thousand (US$11.31 thousand) for small clinics, and 200 thousand (US$6.47 thousand) for medium-scale clinics. The total funding amount for the MCAM in 2019 was $NT 45.10 million (US$ 1.46 million). Once funded, the MMT clinics are asked to expand their manpower by adding one part-time case manager for tiny clinics, one full-time case manager for small clinics, and two full-time case managers for medium-scale clinics.

### Gaps and study aims

Although both the PMES and the MCAM have been implemented since 2019, the impact of these two funding programs on MMT clinics has not yet been rigorously evaluated. Traditional studies have applied randomization or used a control group, which tends to be impractical for public funding programs. A meaningful evaluation of policy interventions poses several methodological challenges, such as the definition of the groups to be compared, the separation of the effect from time to policy, the statistical approaches chosen for evaluation, and the solution to longitudinal correlation within study units [18]. Since the initiation time and the intervention targets of both the PMES and the MCAM are relatively clear, a single interrupted time series analysis (SITSA), which is suitable for a nonrandomized intervention [19], could be used to quantitatively measure the impact of the two policy interventions. In particular, two indices are important in quantifying the efficacy of the policy interventions on MMT clinics, i.e., the monthly average number of daily participants per clinic and the 3-month retention rate. Hence, using SITSA, this study aimed to (1) evaluate the impact of the PMES on the monthly average participants and the 3-month retention rate of MMT clinics from February 2013 to December 2019 and (2) evaluate the impact of the MCAM on the monthly average participants of MMT clinics from September 2019 to December 2019. All analyses were further stratified according to the capacity scale of the MMT clinics.

## Methods

### Outcome of the MMT policy intervention

Two outcome variables for the impact of policy intervention were chosen for this study: the monthly average number of daily participants per clinic, indicating the service quantity, and the 3-month retention rate, indicating the service quality of the MMT clinics.

### Selection of MMT clinics for the current study

Because of the coronavirus disease 2019 (COVID-19) outbreak in 2020, which disrupted the service of MMT clinics in many ways, we decided to examine the data of MMT clinics up to the end of 2019. Among 84 MOHW-contracted MMT clinics, we excluded 6 clinics due to incomplete data during the period of 2013 to 2019 and 3 clinics due to a lack of participants in 2018. In total, 75 MMT clinics with data from February 2013 to December 2019 were included in this study. Due to the partial overlap in time between the PMES and MCAM, the clinics that received funding were separated into (1) PMES only and (2) PMES plus add-on MCAM.

### Data sources and data cleaning processes

For the outcome of the monthly average number of daily participants per clinic, we extracted the monthly number of participants via published statistics from the Department of Mental Health in MOHW from 2013 to 2019. These statistics are released monthly as the number of participants in each of the MMT clinics.

For the outcome of the 3-month retention rate, we reviewed the daily prescription records from the MMT register database, which contains data from 2006 to 2020. If a patient’s MMT prescription records ended without a subsequent record within 14 days, the prescription records within the period comprised one course of treatment. For the estimation of the 3-month retention rate, we extracted treatments with starting dates between February 2013 and December 2019. The calculations were performed by examining the percentage of treatments with a duration ≥ 3 months in all treatments starting in each month. Since the fluctuation of the 3-month retention rate was relatively high, we did not examine the change in the 3-month retention rate for MCAM, which had only 8 monthly points for the pre-intervention period and 4 monthly points for the post-intervention period before the COVID-19 outbreak.

### Statistical analysis

To assess the impact of PMES policy intervention using SITSA, we compared the pre-PMES (2013/02~2018/12) and PMES_12 (2019/01~2019/12) periods for clinics granted by the PMES only. We also compared the pre-PMES (2013/02~2018/12) and PMES_8 (2019/01~2019/08) periods for clinics granted by the PMES and the subsequent add-on MCAM. The impact of the MCAM was evaluated by comparing the periods of PMES_8 (2019/01~2019/08) and PMES_MCAM (2019/09~2019/12) for the clinics granted by PMES and subsequent add-on MCAM.

We applied the following linear regression model for SITSA:

$$y=\beta_0+\beta_1\textit{Time}+\beta_2\textit{Intervention}+\beta_3{\text{Time}}_\textit{intervention}+\varepsilon$$

where (1) y is the outcome in the MMT clinics (e.g., monthly average number of daily participants or 3-month retention rate, aggregating data by all 75 clinics or stratified by different scales), (2) time is the order of time points (e.g., 1, 2, 3, 4 months), with a total of 79 or 83 months in the analysis depending on the periods compared, (3) intervention is the status of policy interventions with time points before the intervention coded as 0 and after the policy intervention coded as 1, and (4) Time_intervention_ is the order of time points after the policy intervention. Time points before the policy intervention are all coded as 0, and time points after the policy intervention are coded as 1, 2, 3, 4, and so on. For the estimates, *β*_1_ is the baseline slope, *β*_2_ is the level change after the policy intervention, and *β*_3_ is the slope change after the policy intervention. All the SITSA models were conducted separately for the overall clinics and tiny-, small-, medium-, and large-scale clinics.

Before the SITSA analysis, possible autocorrelation within data points to the maximum of lag order 12 was examined using the generalized Durbin-Watson test [20, 21]. We chose a Durbin-Watson statistics of < 1.5 as the threshold of having autocorrelation, and the coefficient at the highest lag order before all other coefficients become non-significant (i.e., ≥ 1.5) was selected as the lag order and the corresponding regression standard errors were adjusted for autocorrelation at the identified order using the Newey-West standard errors [22]. We further conducted a sensitivity analysis by removing the data point of December 2014 to examine the influence of outliers in the SITSA. The SITSA was conducted using published SAS codes [23]. All analyses were conducted using SAS version 9.4 (SAS Institute Inc., Cary, NC, USA).

## Results

### Demographic characteristics of MMT clinics in Taiwan

**Fig. 1:**
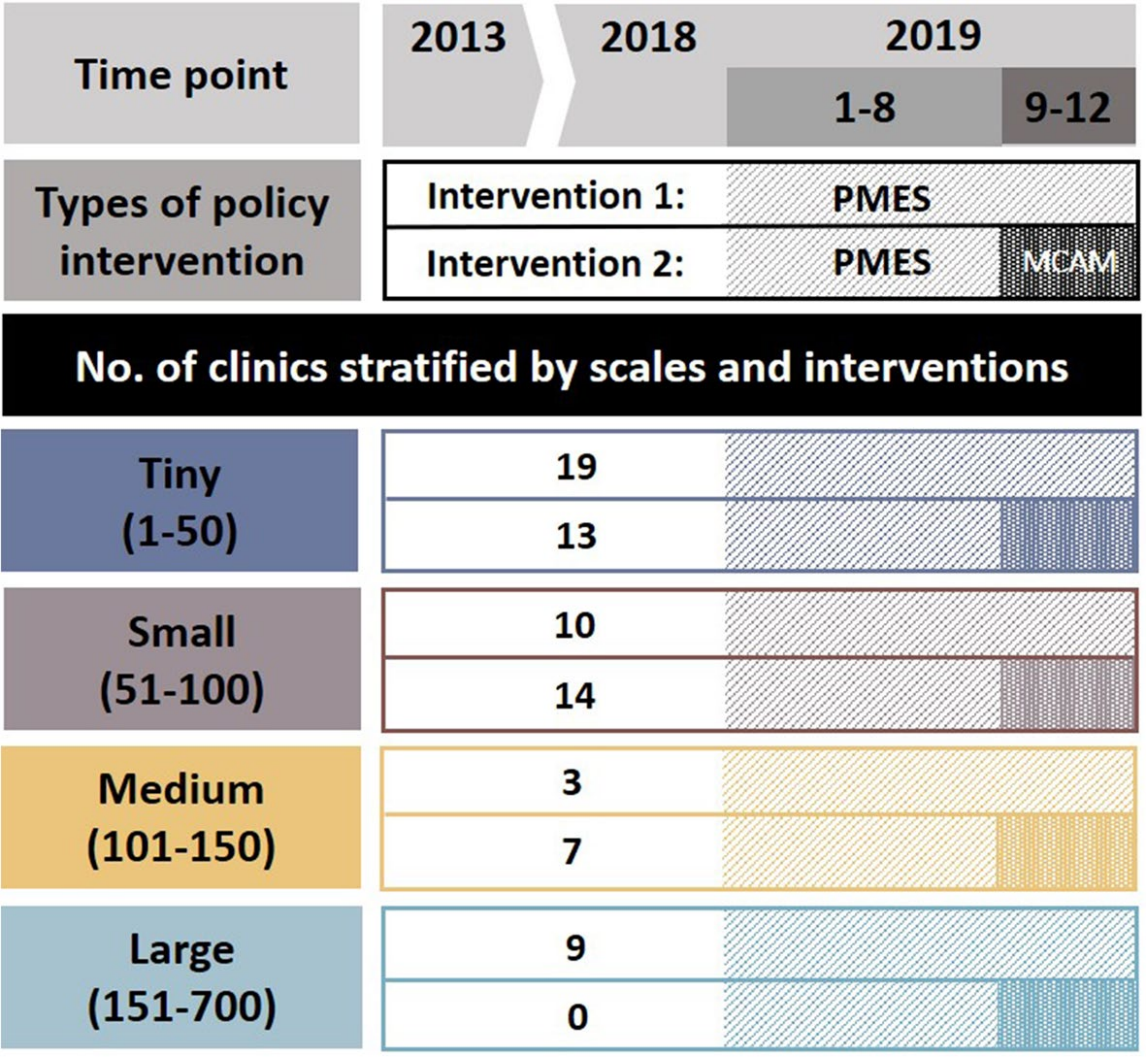
The time frame of two interventions targeting the methadone maintenance treatment (MMT) clinics, i.e., the Patients’ Medical Expenditure Supplement (PMES) program and MMT Clinics Accessibility Maintenance (MCAM) program, and the number of MMT clinics granted for PMES only or for PMES + MCAM were later stratified by the scale of the clinics

The distribution of the MMT clinics at different scales and the timeframe of public funding are depicted in Fig. 1. Among the 75 MMT clinics, 32 were tiny (43%), 24 were small (32%), 10 (13%) were medium, and 9 were large (12%). Their distributions at the facility level, urbanicity, and geographic regions are displayed in Table 1. Most of them were at the hospital level in facility resourcefulness and located in urban areas, whereas their distributions in geographical regions were relatively even, except for the east and outlying islands of Taiwan. After stratification by the scales of capacity, the greater the scale of the MMT clinics, the higher their proportion was in urban areas, ranging from 44% for tiny clinics to 78% for large clinics. Table 1 displays the distributions stratified by whether the PMES was alone, with the distribution similar to that of overall MMT clinics. The geographical regions of the MMT clinics on the map are provided in Supplementary Figure S1.

**Table 1:** Characteristics of methadone maintenance treatment (MMT) clinics at different scales and stratification by government funding programs in Taiwan

	Total MMT (*N* = 75)	By scale				PMES only (*N* = 32)			PMES + MCAM later (*N* = 34)		
		Tiny (*N* = 32)	Small (*N* = 24)	Medium (*N* = 10)	Large (*N* = 9)	Tiny (*N* = 19)	Small (*N* = 10)	Medium (*N* = 3)	Tiny (*N* = 13)	Small (*N* = 14)	Medium (*N* = 7)
Variables	*n* (%)	*n* (%)	*n* (%)	*n* (%)	*n* (%)	*n* (%)	*n* (%)	*n* (%)	*n* (%)	*n* (%)	*n* (%)
Facility level											
Core hospital	22 (29)	7 (22)	8 (33)	3 (30)	4 (44)	5 (26))	2 (20)	1 (33)	2 (15)	6 (43)	2 (29)
Hospital	51 (69)	24 (75)	16 (67)	7 (70)	4 (44)	13 (68)	8 (80)	2 (67)	11 (85)	8 (57)	5 (71)
Clinics	2 (3)	1 (3)	0 (0)	0 (0)	1 (11)	1 (5)	0 (0)	0 (0)	0 (0)	0 (0)	0 (0)
Urbanicity											
Urban	41 (55)	14 (44)	13 (54)	7 (70)	7 (78)	8 (42)	7 (70)	3 (100)	6 (46)	6 (43)	4 (57)
Suburban or rural	34 (45)	18 (56)	11 (46)	3 (30)	2 (22)	11(58)	3 (30)	0 (0)	7 (54)	8 (57)	3 (43)
Geographical region											
North	23 (31)	10 (31)	7 (29)	2 (20)	4 (44)	4 (21)	4 (40)	2 (67)	6 (46)	3 (21)	0 (0)
Central	24 (32)	9 (28)	10 (42)	4 (40)	1 (11)	7 (37)	3 (30)	1 (33)	2 (15)	7 (50)	3 (43)
South	22 (29)	7 (22)	7 (29)	4 (40)	4 (44)	5 (26)	3 (30)	0 (0)	2 (15)	4 (29)	4 (57)
East	4 (5)	4 (13)	0 (0)	0 (0)	0 (0)	1 (5)	0 (0)	0 (0)	3 (23)	0 (0)	0 (0)
Outlying Islands	2 (3)	2 (6)	0 (0)	0 (0)	0 (0)	2 (11)	0 (0)	0 (0)	0 (0)	0 (0)	0 (0)

*PMES* Patients’ Medical Expenditure Supplement program, *MCAM* MMT Clinics Accessibility Maintenance

### The PMES program and the number of participants

**Fig. 2:**
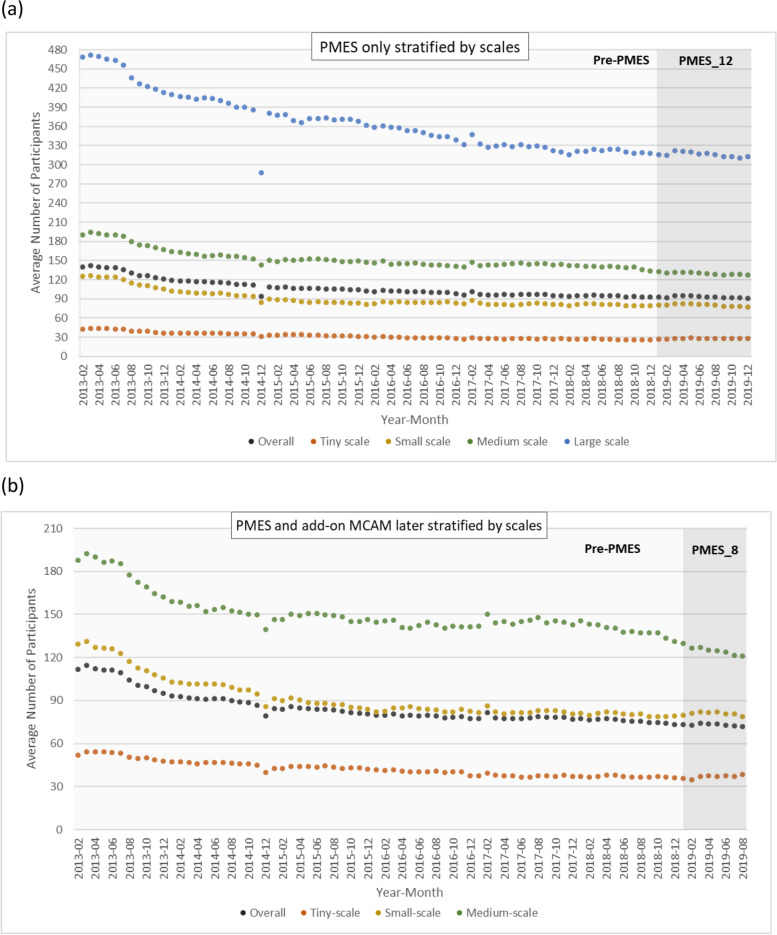
The monthly average number of daily participants per clinic in the periods of **a** pre-PMES and PMES_12 (2019/01~2019/12) in MMT clinics that received PMES only, stratified by the scale of the clinics, and **b** re-PMES and PMES_8 (2019/01~2019/08) in MMT clinics that received PMES and add-on MCAM later, stratified by the scale of the clinics

**Fig. 3:**
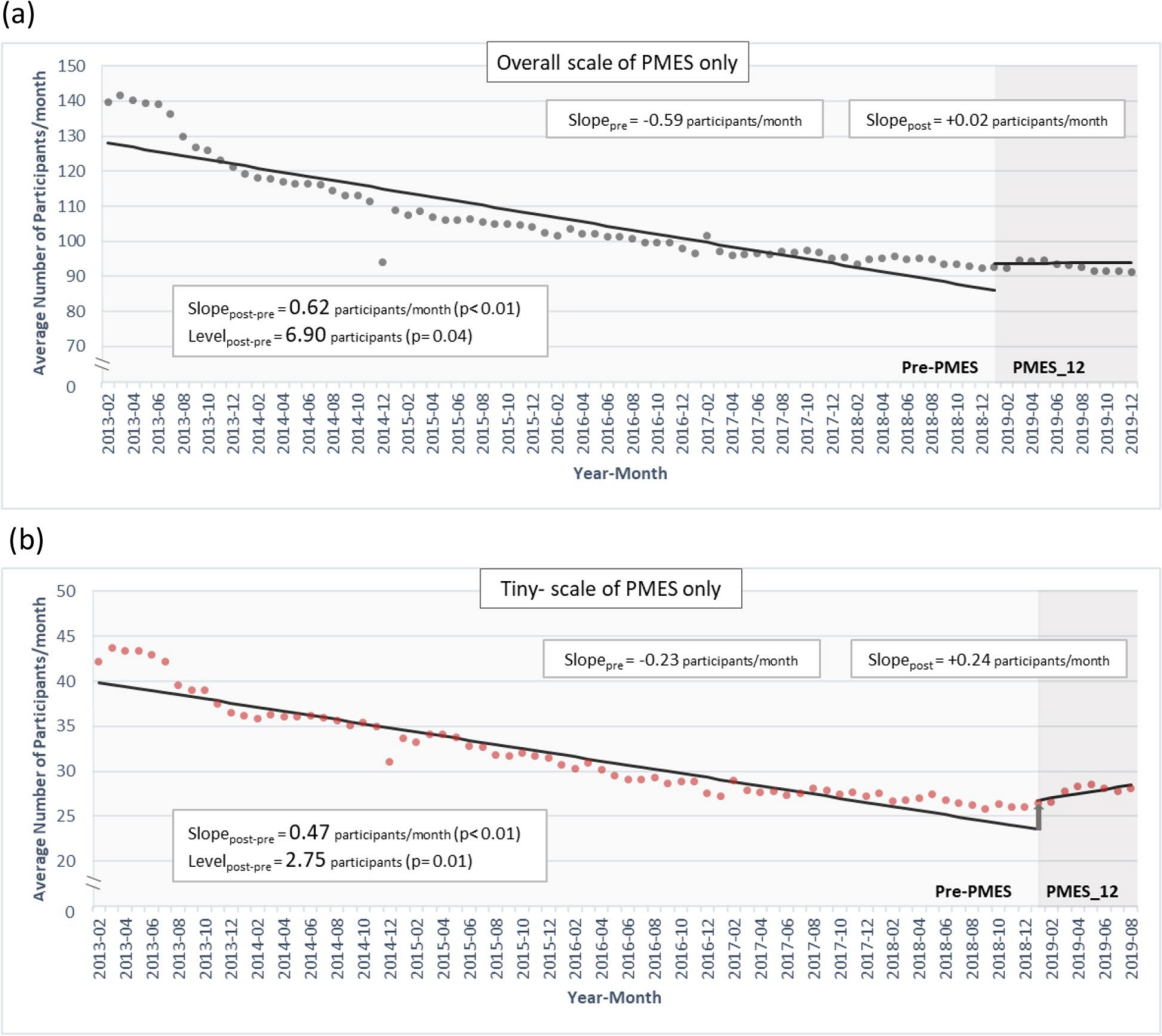
The level change and slope change after the PMES intervention revealed in single interrupted time series analysis of the monthly average number of daily participants per clinic among (**a**) overall clinics that received PMES only and (**b**) tiny-scale clinics that received PMES only

The monthly average number of daily participants per clinic before and after the PMES is shown for MMT clinics at different scales for those receiving PMES only in Fig. 2a and for those receiving PMES with add-on MCAM in Fig. 2b. Based on the Durbin-Watson statistics, the lag parameter within the SITSA for the monthly average number of daily participants for PMES policy intervention was set as 12 and hence Newey-West standard errors were used. Then SITSA was conducted to evaluate the impact of the PMES and the results are displayed in Table 2. For MMT clinics receiving PMES only, before the intervention, there was a decreasing trend in the monthly average number of daily participants in every scale of the MMT clinics, with a baseline slope ranging from − 0.23 to − 1.98 (*p* < 0.01 for all). After the intervention, a significant level elevation and slope increase in the participant number was found in every scale of the MMT clinics except the medium-scale clinics. For the MMT clinics receiving the PMES with add-on MCAM, in which large-scale clinics were not eligible, a preintervention trend of a decreasing monthly average number of daily participants was also found in every scale of the MMT clinics. After the intervention, a level elevation was only found in small-scale clinics, whereas the tiny- and small-scale clinics had a slope increase of 0.29 and 0.62, respectively (*p* < 0.01 for both). Unexpectedly, the medium-scale clinics had a post-intervention slope decrease of 0.64 (*p* < 0.01). For illustration, the impact of the PMES on the monthly average number of daily participants per clinic is depicted for the overall clinics in Fig. 3a and the tiny-scale clinics in Fig. 3b.

**Table 2:** The impact of PMES policy intervention on the monthly average number of daily participants before (non-PMES period) and after the implementation of PMES (PMES period), divided into PMES only (2019/01~2019/12) and PMES with add-on MCAM later (2019/01~2019/08) in Taiwan

Model parameters	PMES only				PMES + add-on MCAM later			
	*n*	β	S.E.^a^	*P* value	*n*	β	S.E.^a^	*P* value
Overall	32				34			
Intercept		128.58	4.78	< 0.01^*^		101.31	4.39	< 0.01^*^
Baseline slope		− 0.59	0.10	< 0.01^*^		− 0.45	0.09	< 0.01^*^
Level change after intervention		6.90	3.38	0.04^*^		4.51	3.01	0.14
Slope change after intervention		0.62	0.12	< 0.01^*^		0.29	0.10	< 0.01^*^
Tiny	19				13			
Intercept		40.35	1.13	< 0.01^*^		51.18	1.21	< 0.01^*^
Baseline slope		− 0.23	0.03	< 0.01^*^		− 0.24	0.03	< 0.01^*^
Level change after intervention		2.75	1.09	0.01^*^		1.02	1.03	0.32
Slope change after intervention		0.47	0.05	< 0.01^*^		0.62	0.04	< 0.01^*^
Small	10				14			
Intercept		110.55	6.11	< 0.01^*^		113.16	5.97	< 0.01^*^
Baseline slope		− 0.55	0.13	< 0.01^*^		− 0.59	0.13	< 0.01^*^
Level change after intervention		9.77	4.34	0.03^*^		10.53	4.50	0.02^*^
Slope change after intervention		0.50	0.16	< 0.01^*^		0.45	0.16	< 0.01^*^
Medium	3				7			
Intercept		174.80	6.83	< 0.01^*^		170.69	7.29	< 0.01^*^
Baseline slope		− 0.61	0.14	< 0.01^*^		− 0.55	0.15	< 0.01^*^
Level change after intervention		1.55	4.28	0.72		−1.04	4.29	0.81
Slope change after intervention		0.15	0.14	0.29		− 0.64	0.15	< 0.01^*^
Large	9				–			
Intercept		438.99	12.27	< 0.01^*^		–	–	–
Baseline slope		− 1.98	0.26	< 0.01^*^		–	–	–
Level change after intervention		19.97	8.51	0.02^*^		–	–	–
Slope change after intervention		1.97	0.32	< 0.01^*^		–	–	–

^a^ Newey-West standard error that is adjusted for autocorrelation

*P value < 0.05

In the sensitivity analysis that removed the data point of December 2014, the results remained almost the same (data not shown). If autocorrelation was not adjusted for in the SITSA, results remained similar except that several slope change estimates failed to reach statistical significance (more details in Supplementary Table S2).

### The PMES program and the 3-month retention rate

**Fig. 4:**
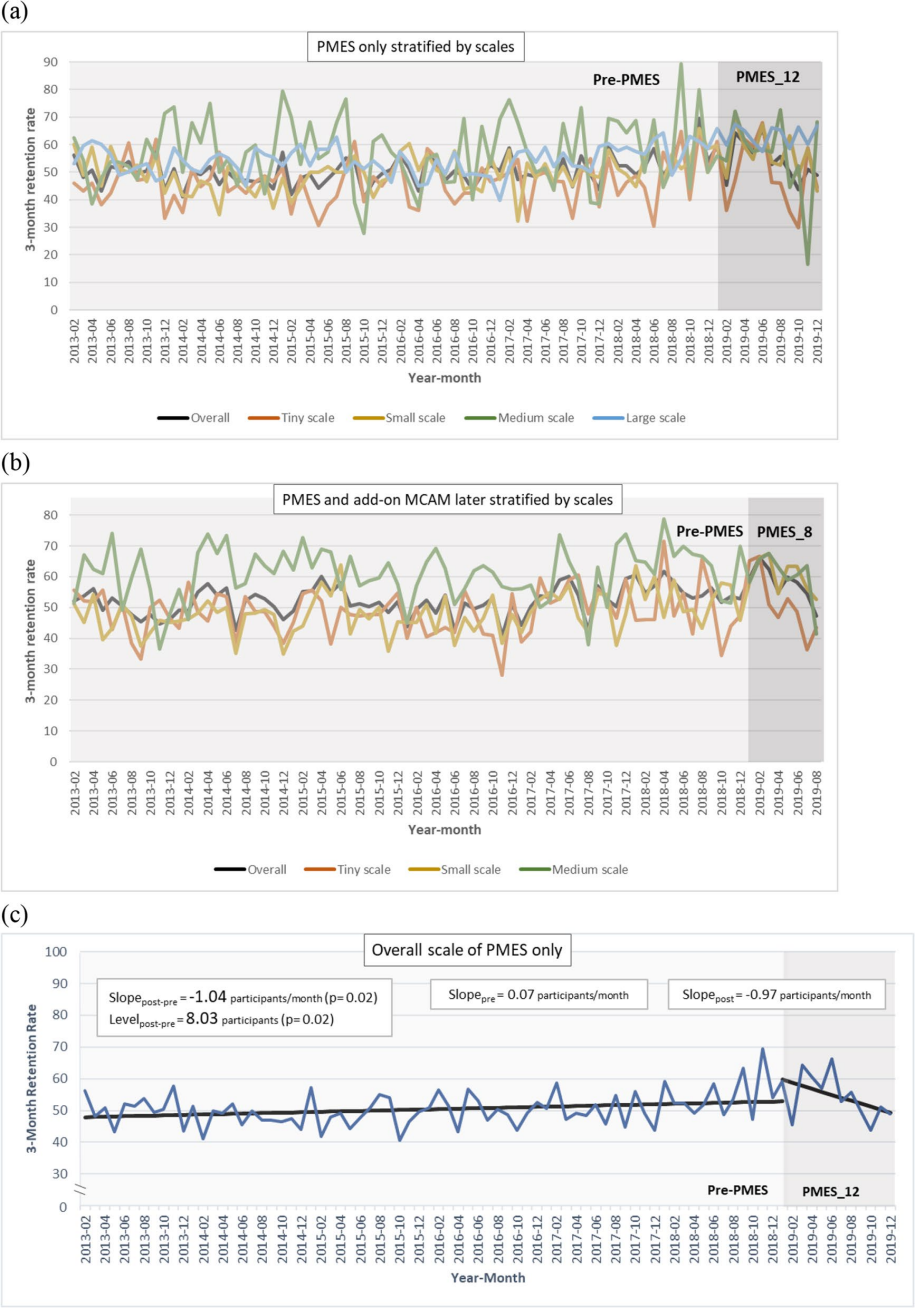
The 3-month retention rate in periods of **a** pre-PMES and PMES_12 (2019/01~2019/12) in MMT clinics that received PMES only, stratified by the scale of the clinics and **b** pre-PMES and PMES_8 (2019/01~2019/08) in MMT clinics that received PMES and add-on MCAM later, stratified by the scale of the clinics and **c** the level change and slope change revealed in single interrupted time series analysis of the 3-month retention rate among overall clinics that received PMES only

The average 3-month retention rate per clinic fluctuated substantially over time in both MMT clinics receiving PMES only (Fig. 4a) and MMT clinics receiving the PMES with the add-on MCAM (Fig. 4b). Based on the Durbin-Watson statistics, the lag parameter within the SITSA for the 3-month retention rate for both PMES and MCAM policy intervention was set as 0, i.e., absence of autocorrelation. When a four-parameter model of SITSA was conducted, the results are shown in Table 3. For MMT clinics receiving PMES only, there was an increasing trend before the intervention in the average 3-month retention rate in the overall MMT clinics (baseline slope = 0.07%, *p* = 0.02), with similar point estimates failing to reach statistical significance in individual scales of MMT clinics. After the intervention, a level elevation in the average 3-month retention rate was found in small- and large-scale as well as overall clinics, whereas a slope decrease in the 3-month retention rate was only found in the overall MMT clinics (slope change = − 1.04%, *p* = 0.02), with similar point estimates failing to reach statistical significance in individual scales of MMT clinics. The impact of the PMES on the trend of 3-month retention rate in overall MMT clinics receiving PMES only is depicted in Fig. 4c.

**Table 3:** The impact of PMES policy intervention on the 3-month retention rate before (non-PMES period) and after the implementation of PMES (PMES period), divided into PMES only (2019/01~2019/12) and PMES with add-on MCAM later (2019/01~2019/08) in Taiwan

Model parameters	PMES only				PMES + add-on MCAM later			
	*n*	β	S.E.	*P* value	*n*	β	S.E.	*P* value
Overall	32				34			
Intercept		47.83	1.26	< 0.01^*^		49.82	1.07	< 0.01^*^
Baseline slope		0.07	0.03	0.02^*^		0.06	0.03	0.02^*^
Level change after intervention		8.03	3.46	0.02^*^		11.78	3.64	< 0.01^*^
Slope change after intervention		− 1.04	0.44	0.02^*^		− 1.87	0.69	< 0.01^*^
Tiny	19				13			
Intercept		44.84	2.07	< 0.01^*^		47.36	1.79	< 0.01^*^
Baseline slope		0.05	0.05	0.36		0.03	0.04	0.42
Level change after intervention		8.27	5.67	0.15		17.84	6.06	< 0.01^*^
Slope change after intervention		− 1.08	0.72	0.14		− 3.65	1.15	< 0.01^*^
Small	10				14			
Intercept		47.23	1.60	< 0.01^*^		44.81	1.47	< 0.01^*^
Baseline slope		0.07	0.04	0.06		0.09	0.04	0.01^*^
Level change after intervention		9.28	4.38	0.04^*^		14.12	5.00	< 0.01^*^
Slope change after intervention		− 0.86	0.56	0.13		− 1.23	0.95	0.20
Medium	3				7			
Intercept		55.58	3.03	< 0.01^*^		59.76	2.03	< 0.01^*^
Baseline slope		0.05	0.07	0.49		0.04	0.05	0.44
Level change after intervention		5.51	8.31	0.51		5.66	6.89	0.41
Slope change after intervention		− 1.39	1.06	0.19		− 1.86	1.31	0.16
Large	9				–			
Intercept		52.77	1.16	< 0.01^*^		–	–	–
Baseline slope		0.04	0.03	0.15		–	–	–
Level change after intervention		7.78	3.19	0.02^*^		–	–	–
Slope change after intervention		− 0.05	0.41	0.90		–	–	–

*P value < 0.05

For MMT clinics receiving the PMES with the add-on MCAM, also shown in Table 3, there was an increasing trend in the 3-month retention rate before the intervention overall (baseline slope = 0.06%, *p* = 0.02) and small-scale (baseline slope = 0.09%, *p* = 0.01) clinics. After the intervention, a level elevation in the average 3-month retention rate was found at every eligible scale (11.78% overall, 17.84% for tiny, and 14.12% for the small-scale clinics) except the medium-scale clinics, whereas a slope decrease in the 3-month retention rate was found in overall (− 1.87%, *p* < 0.01) and tiny-scale (− 3.65%, *p* < 0.01) clinics.

### The MCAM program and the number of participants

**Fig. 5:**
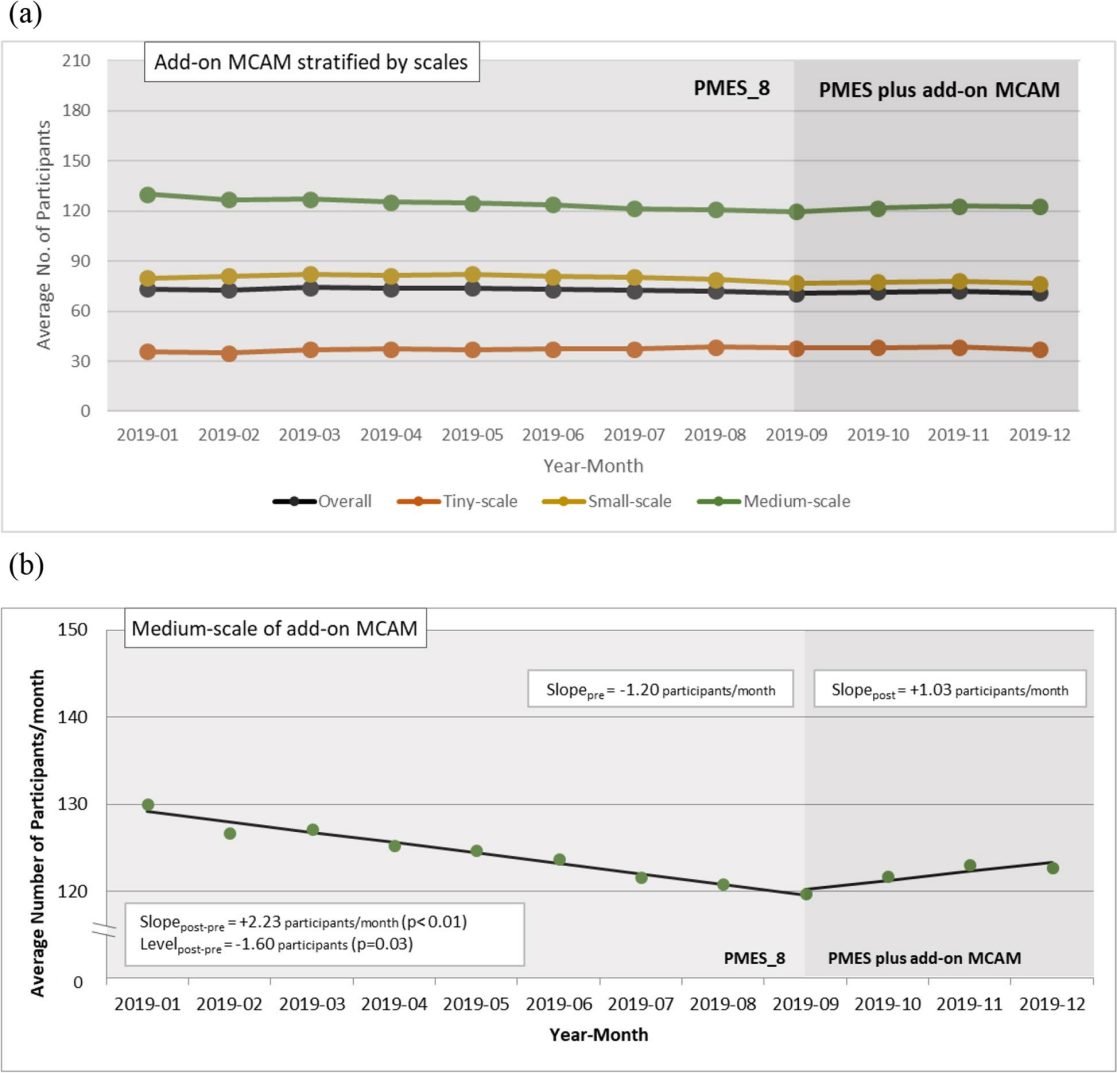
The impact of the add-on MCAM: **a** the monthly average number of daily participants per clinic in the periods of PMES_8 (2019/01~2019/08) and PMES plus add-on MCAM (2019/09~2019/12) in those MMT clinics that received MCAM, stratified by the scale of the clinics, and **b** the level change and slope change revealed in single interrupted time series analysis of the monthly average number of daily participants per clinic among medium-scale MMT clinics

For MMT clinics that received the MCAM program, the monthly average number of daily participants per clinic during the study period of 2019 is shown in Fig. 5a. Based on the Durbin-Watson statistics, the lag parameter within the SITSA for the monthly average number of daily participants for MCAM policy intervention was set as 0 for overall and tiny-scale clinics and as 1 for small- and medium-scale clinics, in which Newey-West standard errors were adopted. The results of the SITSA on the impact of add-on MCAM are displayed in Table 4. Before the intervention, there was an increasing trend in tiny-scale clinics (baseline slope = 0.38, *p* = 0.01) but a decreasing trend in medium-scale clinics (baseline slope = − 1.20, *p* < 0.01). After the intervention, the impact was found in medium-scale clinics, with a slope increase of 2.23 (*p* < 0.01) accompanied by a level decrease of 1.60 (*p* = 0.03), and in small-scale clinics, with a level decrease of 3.11 (*p* < 0.01). The upturn in the slope after the implementation of the MCAM is depicted in Fig. 5b. If autocorrelation was not adjusted for in the SITSA for small- and medium-scale clinics, the level decrease after intervention became non-significant in these clinics (more details in Supplementary Table S3).

**Table 4:** The impact of the add-on MCAM policy intervention on the monthly average number of daily participants before (PMES-only period) and after the implementation of MCAM (PMES with add-on MCAM period) from September 2019 to December 2019 in Taiwan

Model parameters	PMES + add-on MCAM			
	*n*	β	S.E.^a^	*P* value
Overall	34			
Intercept		73.86	0.54	< 0.01^*^
Baseline slope		− 0.16	0.11	0.16
Level change after intervention		− 1.50	0.95	0.15
Slope change after intervention		0.28	0.33	0.41
Tiny	13			
Intercept		35.19	0.53	< 0.01^*^
Baseline slope		0.38	0.10	0.01^*^
Level change after intervention		0.29	0.94	0.76
Slope change after intervention		− 0.62	0.32	0.09
Small	14			
Intercept		81.50	0.99	< 0.01^*^
Baseline slope		− 0.15	0.19	0.46
Level change after intervention		− 3.11	0.72	< 0.01^*^
Slope change after intervention		0.14	0.30	0.65
Medium	7			
Intercept		130.39	0.42	< 0.01^*^
Baseline slope		− 1.20	0.07	< 0.01^*^
Level change after intervention		− 1.60	0.60	0.03^*^
Slope change after intervention		2.23	0.22	< 0.01^*^

^a^Using Newey-West standard errors that are adjusted for autocorrelation for small- and medium-scale clinics

*P value < 0.05

## Discussion

In the current study, we applied SITSA to examine whether the two public funding programs, the PMES and MCAM, helped to alleviate participant reduction and improve the 3-month retention rate among MMT clinics in Taiwan, which is one of the few Asian countries that have implemented a nationwide harm-reduction program. Before the intervention, MMT clinics exhibited a decreasing trend in participant numbers but an increasing trend in the 3-month retention rate over time across every scale of the clinics. The implementation of the PMES led to a level increase and slope increase in the participant numbers at every scale of clinics that solely received the PMES except the medium-scale clinics. Nevertheless, a post-PMES level elevation in participant numbers was only seen in small-scale clinics that applied for MCAM later. Although the 3-month retention rate had a post-PMES level elevation at every scale except medium-scale clinics, it was accompanied by a slope decrease in overall and tiny-scale clinics. For MMT clinics that had received the PMES in the beginning of 2019 and received further funding from the MCAM in the last 4 months of 2019, we observed a pre-MCAM increasing trend in participant numbers (i.e., a post-PMES slope increase) in tiny-scale clinics but a post-MCAM slope increase in participant numbers in medium-scale clinics. Our results demonstrate differential efficacies of public funding programs on MMT clinics of varying capacities and have implications for future refinement.

Applying SITSA to the registry data of the MMT clinics before the intervention allows us to quantify the speed of participant reduction and to identify, surprisingly, an improving trend in the 3-month retention rate over time. One possible explanation for the pre-PMES declining participant numbers was the new option of buprenorphine treatment that began to be offered nationwide in 2010. The consumption of buprenorphine as a substitute for heroin in maintenance programs in defined daily doses for statistical purposes per million inhabitants per day (S-DDD/m/d) increased rapidly from 7.6 in 2010 to 26.2 in 2011 and 72.4 in 2014 [24]. A second possible explanation is the decreasing trend in the number of first-time offenders of heroin use after the implementation of the nationwide harm reduction program, with the number decreasing from 1257 in 2005 to 821 in 2006 and further to 69 in 2017 [25]. The declining number of participants might inadvertently make those enrolled in the MMT clinics more likely to be patients with higher motivation for treatment and hence improve the 3-month retention rate.

Many factors might contribute to the post-PMES level elevation in the participant numbers. A major reason is that the PMES helped to diminish the financial burden of attending or continuing the MMT program since cost was often perceived as the major obstacle by people with heroin use [26]. In particular, the additional cost of a broad treatment repertoire that included psychosocial intervention [27], which helps to relieve discomfort in MMT [28] and prevent patient drop-out [29], greatly limited people with heroin use access to these treatments. Our results extend beyond an earlier finding from a cross-sectional survey in MMT clinics in China that clinics providing more than two types of comprehensive services were associated with more participants [30]. With financial support from the PMES for these treatments, more people with heroin use were willing to be enrolled in MMT.

Regarding the 3-month retention rate, which was evaluated only for the PMES, we found a post-PMES level elevation at every scale of the clinics except medium-scale clinics. This might be accounted for by the PMES’s supplement for case management and outreach services, which allowed MMT clinics to have more human resources to maintain frequent connection with patients and help decrease patients’ loss of contact, which is the most common reason for interruption in MMT [31]. This is consistent with the extant literature, in which 6-week outreach case management was found to be nearly six times more likely to reengage patients compared to passive referral [32]. Furthermore, a recent meta-analysis concluded that case management effectively improves the linkage and retention of treatment [33]. Nevertheless, the level elevation in the 3-month retention rate was accompanied by a slope decrease in overall and tiny-scale clinics. This may be caused by the inadequacy in human resources to keep up with the increasing demand in case management when more patients remain in MMT clinics.

Nevertheless, our analyses also revealed that 20 out of 66 clinics that received the PMES failed to show a substantial post-PMES level elevation in the participant numbers. When these 20 clinics received further funding from the MCAM, a post-MCAM slope increase in the participant numbers was seen only in the medium-scale clinics. This might be related to the request of the MCAM that funded MMT clinics needed to have two full-time case managers for medium-scale clinics but one full-time case manager for small-scale clinics and a part-time case manager for tiny-scale clinics. This would make the average caseload per full-time case manager lower for medium-scale clinics (101–150 participants per month) than for small-scale clinics (51–100 participants per month).

### Public health implications

Our findings about the differential efficacies of two public funding programs in temporarily increasing the participant numbers and improving the 3-month retention rate have implications for future improvement. First, supplementation of the MMT-related cost, including the additional cost for psychosocial intervention to broaden the treatment repertoire, is essential to reduce the obstacles for people with heroin use to attend and continue MMT, as illustrated by the post-PMES level elevation in the participant numbers. Second, sufficient manpower, particularly case managers who have a reasonable caseload, is essential to keep up with the increasing numbers of participants and 3-month retention rate, as illustrated by the post-MCAM slope increase in the participant numbers and the post-PMES level increase in the 3-month retention rate. Without sufficient manpower, solely supplementing patients’ medical expenditure can only lead to a temporary increase in the participant numbers or 3-month retention rate (i.e., a postintervention level increase) that is likely to be accompanied by a decreasing trend thereafter (i.e., a postintervention slope decrease).

Another challenge for MMT clinics is how to balance tight supervision to ensure compliance and affordable flexibility to increase accessibility, as illustrated by the government’s responses to the disruption of MMT services during the COVID-19 pandemic in different countries. In Taiwan, the government set up satellite stations for MMT to improve accessibility in suburban and rural areas [34]. In the USA, the federal government loosened regulations for take-home methadone doses and increased the availability of telehealth, resulting in a challenge for care providers to both minimize harm and help advance the treatment of opioid use disorder [35, 36]. It is important for new approaches after COVID-19, such as the fee for taking methadone home, to be covered by public funding to sustain accessibility [37].

To maintain or enhance the accessibility and retention of people with heroin use at MMT clinics in the future, the government needs to supplement both patients’ costs and institutions’ manpower and adopt a new balance between tight supervision and practical flexibility by allowing for take-home dosing and telehealth.

## Limitations

There are some limitations to this study. First, due to the interruption of MMT service by the COVID-19 pandemic, the study time period covered by the two funding programs was truncated before the outbreak of COVID-19 in Taiwan. The time points after the postintervention might not be long enough for certain outcome evaluations, particularly for the MCAM (only 4 monthly points). Second, this study’s evaluation of the implementation process of the two policy interventions was limited to the information available in the current public database. Third, there was no information about the reliability and accuracy of the MMT registry and the published statistics by the MOHW. Nevertheless, as a schedule II controlled drug, methadone is produced and delivered solely by the Taiwan Food and Drug Administration (TFDA) in the MOHW. Any medical institution that requires methadone for treatment needs to obtain approval from TFDA first, record the amount of consumption and amount in stock every day, and provide regular reports to the local government and TFDA. Meanwhile, the local government regularly visits the contracted MMT clinics to audit the reliability of the reports from the medical institution. All these rigorous measures by the government may decrease the potential missingness and increase the reliability and accuracy of the MMT registry database. Fourth, the exact items in eligible categories for supplementation chosen by individual MMT clinics varied. Due to the lack of relevant information from individual MMT clinics, their PMES might be compared with different compositions. Finally, since this study was exploratory in nature, we did not adjust for multiple testing in our analyses.

## Conclusions

By supplementing the cost of a broad treatment repertoire, the PMES removed barriers for people with heroin use to attend MMT clinics, resulting in a temporary increase in the number of participants and the 3-month retention rate. The MCAM exerted its impact by increasing investment in human resources, which is more relevant for clinics on a larger scale. Hence, supplementing the cost of a broad treatment repertoire enhances the enrollment of people with heroin use in MMT, and further funding of human resources is vital for MMT clinics to keep up with the increasing number of participants and their retention.

### Supplementary Information

**Additional file 1: Figure S1.** Geographic distribution of the MMT clinics granted for PMES only or for PMES + MCAM later stratified by the scale of the clinics. **Table S1.** Subsidized items in the PMES since 2019. **Table S2.** Complete parameters of models, without autocorrelation adjustment, evaluating the impact of PMES policy intervention on the monthly average number of daily participants comparing the periods of non-PMES vs. PMES, which were divided into PMES only (2019/01~2019/12) and PMES with add-on MCAM later (2019/01~2019/08). **Table S3.** Complete parameters of models, without autocorrelation adjustment, evaluating the impact of further MCAM policy intervention on the monthly average number of daily participants comparing the periods of pre-MCAM (i.e., PMES only, 2019/01~2019/08) vs. post-MCAM (i.e., PMES+MCAM, 2019/09~2019/12).

## Data Availability

Data are available upon request from the corresponding author.

## Abbreviations

HIV: Human immunodeficiency virus
NSP: Needle-syringe program
OST: Opioid substitution therapy
MMT: Methadone maintenance treatment
MOHW: Ministry of health and welfare
PMES: Medical expenditure supplements
MCAM: MMT clinics accessibility maintenance
SITSA: Single interrupted time series analysis

## References

[1] United Nations Office on Drugs and Crime (2021). World Drug Report 2021. 1. Executive Summary, Policy Implications.

[2] United Nations Office on Drugs and Crime (2022). World Drug Report 2022. 1. Executive Summary, Policy Implications.

[3] Connery HS (2015). Medication-assisted treatment of opioid use disorder: review of the evidence and future directions. Harv Rev Psychiatry.

[4] Thomson N (2013). Harm reduction history, response, and current trends in Asia. J Food Drug Anal.

[5] Li J-H (2012). Evolution of the legislative and administrative system of controlled drugs in Taiwan. J Food Drug Anal.

[6] Bell J (2010). The global diversion of pharmaceutical drugs. Opiate treatment and the diversion of pharmaceutical opiates: a clinician's perspective. Addiction.

[7] Khatapoush S, Hallfors D (2004). “Sending the wrong message”: Did medical marijuana legalization in California change attitudes about and use of marijuana?. J Drug Issues.

[8] Lin T, Chen CH, Chou P (2016). Effects of combination approach on harm reduction programs: the Taiwan experience. Harm Reduct J.

[9] Chen JS (2011). Beyond human rights and public health: citizenship issues in harm reduction. Int J Drug Policy.

[10] Department of Mental Health. Statistical reports of maintenance treatment (https://dep.mohw.gov.tw/DOMHAOH/lp-4223-107-4-20.html). In*.* Taipei: Department of Mental Health, Ministry of Health and Welfare; 2017.

[11] Chang KC, Lee KY, Lu TH, Hwang JS, Lin CN, Ting SY, Chang CC, Wang JD (2019). Opioid agonist treatment reduces losses in quality of life and quality-adjusted life expectancy in heroin users: Evidence from real world data. Drug Alcohol Depend..

[12] Huang C-L, Tsai IJ, Lin W-C, Lin C-L, Ho I-K, Wang R-Y, Lee CW-S (2021). Reduced mortality in patients with extended duration of methadone maintenance treatment: a five-year retrospective nationwide study. Psychol Med.

[13] Bullock HL, Lavis JN, Wilson MG, Mulvale G, Miatello A (2021). Understanding the implementation of evidence-informed policies and practices from a policy perspective: a critical interpretive synthesis. Implement Sci.

[14] Chen Y-MA, Kuo SH-S (2007). HIV-1 in Taiwan. Lancet.

[15] Mathers BM, Degenhardt L, Ali H, Wiessing L, Hickman M, Mattick RP, Myers B, Ambekar A, Strathdee SA (2010). HIV prevention, treatment, and care services for people who inject drugs: a systematic review of global, regional, and national coverage. Lancet.

[16] Executive Yuan. Government at a glance: National Health Insurance. 2023. https://www.ey.gov.tw/state/A01F61B09E09A9758D/fa9706e9750d9752-9413f-9401e-b9694-9720c9752db9786f9404. Accessed 25 Jan 2024.

[17] Directorate-General of Budget Accounting and Statistics at Executive Yuan (2019). Report on the survey of family income and expenditure.

[18] French B, Heagerty PJ (2008). Analysis of longitudinal data to evaluate a policy change. Stat Med.

[19] Brown CH, Curran G, Palinkas LA, Aarons GA, Wells KB, Jones L, Collins LM, Duan N, Mittman BS, Wallace A (2017). An overview of research and evaluation designs for dissemination and implementation. Annu Rev Public Health.

[20] Durbin J, Watson GS (1950). Testing for serial correlation in least squares regression, I. Biometrika.

[21] Durbin J, Watson GS (1951). Testing for serial correlation in least squares regression, II. Biometrika.

[22] Newey WK, West KD (1987). A simple, positive semi-definite, heteroskedasticity and autocorrelation consistent covariance matrix. Econometrica.

[23] Caswell J. Interrupted time series analysis for single series and comparative designs: A guide for beginners with SAS Macro. Updated on 2019. Available online https://www.academia.edu/35275583/Interrupted_Time_Series_Analysis_for_Single_Series_and_Comparative_Designs_A_Guide_for_Beginners_with_SAS_Macro. Accessed 30 Oct 2023.

[24] Kang K-H, Kuo L-F, Cheng IC, Chang C-S, Tsay W-I (2017). Trends in major opioid analgesic consumption in Taiwan, 2002–2014. J Formos Med Assoc.

[25] Chen WJ, Chen C-Y, Wu S-C, Wu KC-C, Jou S, Tung Y-C, Lu T-P (2021). The impact of Taiwan’s implementation of a nationwide harm reduction program in 2006 on the use of various illicit drugs: trend analysis of first-time offenders from 2001 to 2017. Harm Reduct J.

[26] Khazaee-Pool M, Moeeni M, Ponnet K, Fallahi A, Jahangiri L, Pashaei T (2018). Perceived barriers to methadone maintenance treatment among Iranian opioid users. Int J Equity Health.

[27] Deering DEA, Sheridan J, Sellman JD, Adamson SJ, Pooley S, Robertson R, Henderson C (2011). Consumer and treatment provider perspectives on reducing barriers to opioid substitution treatment and improving treatment attractiveness. Addict Behav.

[28] Philbin MM, Zhang F (2010). Exploring stakeholder perceptions of facilitators and barriers to accessing methadone maintenance clinics in Yunnan Province, China. AIDS Care.

[29] Fan X, Zhang X, Xu H, Yang F, Lau JTF, Hao C, et al. Effectiveness of a psycho-social intervention aimed at reducing attrition at methadone maintenance treatment clinics: A propensity score matching analysis. Int J Environ Res Public Health. 2019;16(22). 10.3390/ijerph16224337 PMC6888175 31703302

[30] Lin C, Wu Z, Rou K, Yin W, Wang C, Shoptaw S, Detels R (2010). Structural-level factors affecting implementation of the methadone maintenance therapy program in China. J Subst Abuse Treat.

[31] Andraka-Christou B, Totaram R, Nguyen TD (2022). Comprehensive analysis of discharge reasons from methadone outpatient treatment programs. Am J Addict.

[32] Coviello DM, Zanis DA, Wesnoski SA, Alterman AI (2006). The effectiveness of outreach case management in re-enrolling discharged methadone patients. Drug Alcohol Depend.

[33] Vanderplasschen W, Rapp RC, De Maeyer J, Van Den Noortgate W (2019). A meta-analysis of the efficacy of case management for substance use disorders: A recovery perspective. Front Psychiatry.

[34] Shih CC, Chen Y, Shao SC, Lai EC (2020). Strategies to maintain persistence of opioid agonist therapy during the novel coronavirus pandemic in Taiwan. Drug Alcohol Depend.

[35] Leppla IE, Gross MS (2020). Optimizing medication treatment of opioid use disorder during COVID-19 (SARS-CoV-2). J Addict Med.

[36] Brothers S, Palayew A, Simon C, Coulter A, Strichartz K, Voyles N, Vincent L (2023). Patient experiences of methadone treatment changes during the first wave of COVID-19: a national community-driven survey. Harm Reduct J.

[37] Suen LW, Coe WH, Wyatt JP, Adams ZM, Gandhi M, Batchelor HM, Castellanos S, Joshi N, Satterwhite S, Pérez-Rodríguez R (2022). Structural adaptations to methadone maintenance treatment and take-home dosing for opioid use disorder in the era of COVID-19. Am J Public Health.

