# Correction: Assessing the impact of public funding in alleviating participant reduction and improving the retention rate in methadone maintenance treatment clinics in Taiwan: an interrupted time series analysis

**DOI:** 10.1186/s13012-024-01358-8

**Published:** 2024-03-19

**Authors:** Yu-Chu Ella Chung, Yu-Chi Tung, Sheng-Chang Wang, Chieh-Liang Huang, Lian-Yu Chen, Wei J. Chen

**Affiliations:** 1 Center for Neuropsychiatric Research, National Health Research Institutes, Miaoli, Taiwan; 2 Institute of Health Policy and Management, College of Public Health, National Taiwan University, Taipei, Taiwan; 3 Tsaotun Psychiatric Center, Ministry of Health and Welfare, Nan-Tou County, Taiwan; 4 Department of Mental Health, Ministry of Health and Welfare, Taipei, Taiwan; 5 Institute of Epidemiology and Preventive Medicine, College of Public Health, National Taiwan University, Taipei, Taiwan; 6 Department of Public Health, College of Public Health, National Taiwan University, Taipei, Taiwan; 7 Department of Psychiatry, College of Medicine and National Taiwan University Hospital, National Taiwan University, Taipei, Taiwan

**Correction** **:** **Implement Sci 19, 18 (2024)**

**https://doi.org/10.1186/s13012-024-01351-1**

Following the publication of the original article [1], the authors reported errors in Figure 1 and in a line of Page 3. In the original article, Figure 1 is shown as below.


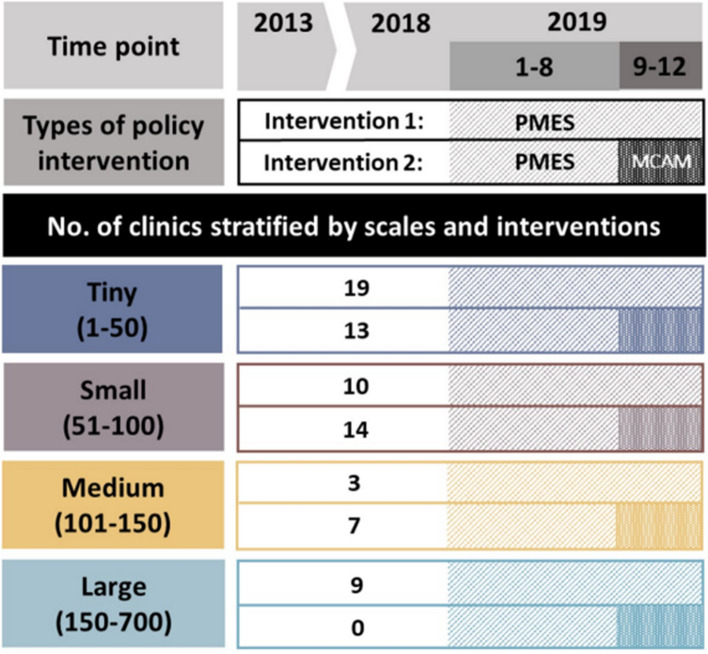


There was an error with regard to the Large number (150-700); as it should have been (151-700).

Thus, the correct Figure 1 is shown below:


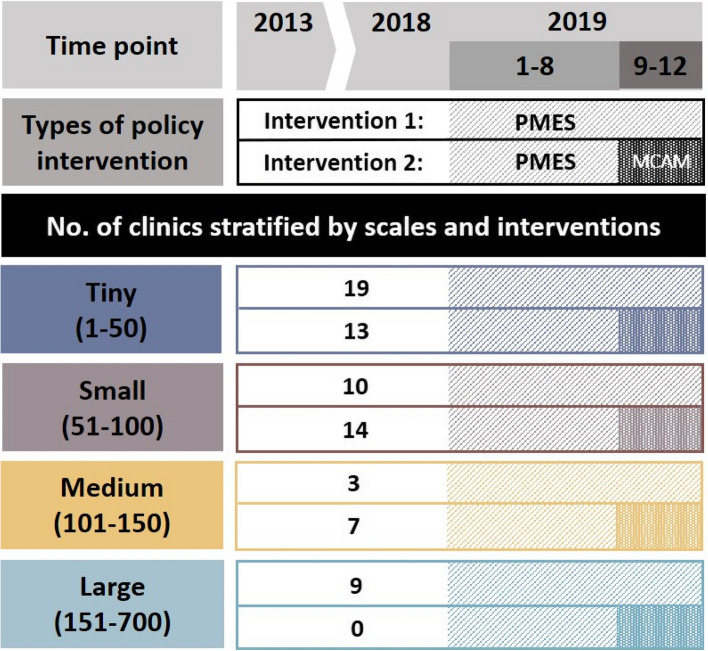


Another error was also found on Page 3 of the Original Article. The second sentence of the second paragraph of the *Context of the MMT policy intervention* section was read “For MMT clinics with larger capacities that were not eligible for the MCAM, we designated their scale as “large”, i.e., monthly average number of daily participants of 150 to 700.”

The correct sentence is: For MMT clinics with larger capacities that were not eligible for the MCAM, we designated their scale as “large”, i.e., monthly average number of daily participants of 151 to 700.

The original article [1] has been updated.
