# Supplementary material for: Assessing the impact of public funding in alleviating participant reduction and improving the retention rate in methadone maintenance treatment clinics in Taiwan: an interrupted time series analysis
Source: Implement Sci. 2024 Feb 22;19:18. doi: 10.1186/s13012-024-01351-1 (PMC10885479; doi:10.1186/s13012-024-01351-1)
Supplement: Supplementary file 1 — Additional file 1: Figure S1. Geographic distribution of the MMT clinics granted for PMES only or for PMES + MCAM later stratified by the scale of the clinics. Table S1. Subsidized items in the PMES since 2019. Table S2. Complete parameters of models, without autocorrelation adjustment, evaluating the impact of PMES policy intervention on the monthly average number of daily participants comparing the periods of non-PMES vs. PMES, which were divided into PMES only (2019/01~2019/12) and PMES with add-on MCAM later (2019/01~2019/08). Table S3. Complete parameters of models, without autocorrelation adjustment, evaluating the impact of further MCAM policy intervention on the monthly average number of daily participants comparing the periods of pre-MCAM (i.e., PMES only, 2019/01~2019/08) vs. post-MCAM (i.e., PMES+MCAM, 2019/09~2019/12). [file 13012_2024_1351_MOESM1_ESM.docx]

**Supplementary materials**

**Supplementary figures**

Figure S1. Geographic distribution of the MMT clinics granted for PMES only or for PMES + MCAM later stratified by the scale of the clinics.

**Supplementary tables**

Table S1. Subsidized items in the PMES since 2019.

Table S2. Complete parameters of models, without autocorrelation adjustment, evaluating the impact of PMES policy intervention on the monthly average number of daily participants comparing the periods of non-PMES vs. PMES, which were divided into PMES only (2019/01~2019/12) and PMES with add-on MCAM later (2019/01~2019/08).

Table S3. Complete parameters of models, without autocorrelation adjustment, evaluating the impact of further MCAM policy intervention on the monthly average number of daily participants comparing the periods of pre-MCAM (i.e., PMES only, 2019/01~2019/08) vs. post-MCAM (i.e., PMES+MCAM, 2019/09~2019/12).


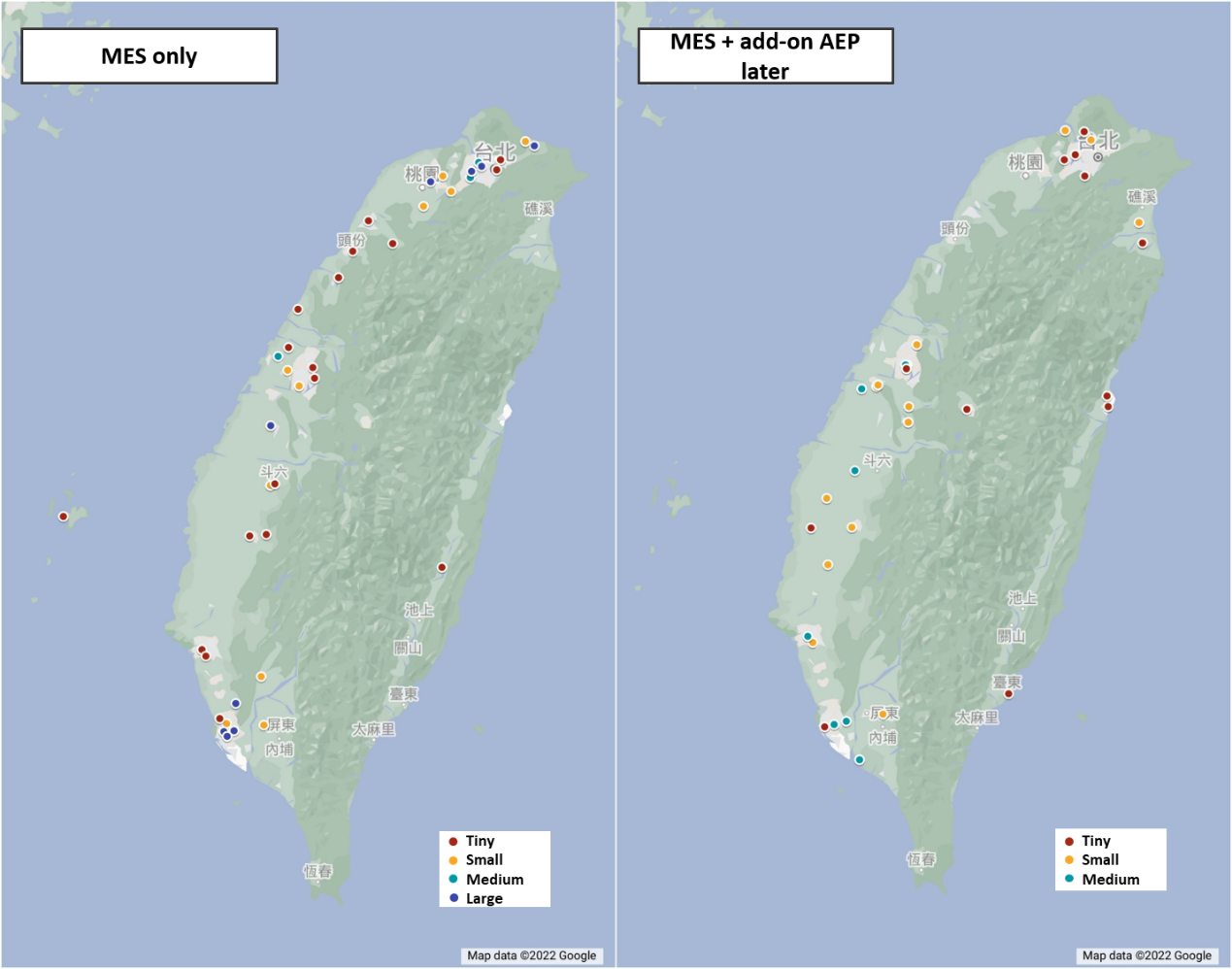


Figure S1. Geographic distribution of the MMT clinics granted for PMES only or for PMES + MCAM later stratified by the scale of the clinics.

Table S1. Subsidized items in the PMES since 2019

| **Items** | **Amount of subsidy** |
| --- | --- |
| Fee for assessment at outpatient clinic | 338 NTD/time |
| Fee for assessing physiological and psychological function | ≥18-year old：344 NTD/time  <18-year old：387 NTD/time |
| Fee for diagnostic interview for drug use disorder | ≥18-year old：1,237 NTD/time  <18-year old：1,444 NTD/time |
| Fee for assessing social functioning | 413 NTD/time |
| Fee for psychological assessment | 1,650 NTD/time |
| Fee for occupational assessment for drug use disorder | 824 NTD/time |
| Fee for supportive interview for drug use disorder | 116 NTD/time |
| Fee for individual psychotherapy for drug use disorder | ≥18-year old：1,444 NTD/time  <18-year old：1,752 NTD/time |
| Fee for group therapy for drug use disorder | Either one:  1.Subsidize patient：420 NTD/time/person  2. Subsidize leader for group therapy：1,800 NTD/hour |
| Fee for family therapy for drug use disorder | 1,200 NTD/time |
| Fee for occupational therapy for drug use disorder | 390 NTD/time |
| Fee for urine drug testing | 300 NTD/time |
| Fee for psychiatric nursing care for mental illness | 129 NTD/day |
| Fee for psychiatric inpatient care | 1,547 NTD/day |
| Fee for case management for patient with drug use | 150 NTD/time |
| Fee for outreach service for patient with drug use | 500 NTD/time |
| Fee for liaison consultation | 409 NTD/time |

Table S2. Complete parameters of models, without autocorrelation adjustment, evaluating the impact of PMES policy intervention on the monthly average number of daily participants comparing the periods of non-PMES vs. PMES, which were divided into PMES only (2019/01~2019/12) and PMES with add-on MCAM later (2019/01~2019/08).

| **Parameters in the model** | **PMES only** | | | |  | **PMES + add-on MCAM later** | | | |
| --- | --- | --- | --- | --- | --- | --- | --- | --- | --- |
|  | **n** | **β** | **S.E.** | **P‐value** |  | **n** | **β** | **S.E.** | **P‐value** |
| **Overall** | 32 |  |  |  |  | 34 |  |  |  |
| Intercept |  | 128.58 | 1.30 | <0.01^*^ |  |  | 101.31 | 1.18 | <0.01^*^ |
| Baseline slope |  | -0.59 | 0.03 | <0.01^*^ |  |  | -0.45 | 0.03 | <0.01^*^ |
| Level change after intervention |  | 7.78 | 3.57 | 0.03^*^ |  |  | 4.51 | 4.01 | 0.26 |
| Slope change after intervention |  | 0.37 | 0.45 | 0.42 |  |  | 0.29 | 0.76 | 0.71 |
| **Tiny** | 19 |  |  |  |  | 13 |  |  |  |
| Intercept |  | 40.35 | 0.35 | <0.01^*^ |  |  | 51.18 | 0.39 | <0.01^*^ |
| Baseline slope |  | -0.23 | 0.01 | <0.01^**^ |  |  | -0.24 | 0.01 | <0.01^*^ |
| Level change after intervention |  | 3.20 | 0.96 | <0.01^**^ |  |  | 1.02 | 1.34 | 0.45 |
| Slope change after intervention |  | 0.35 | 0.12 | 0.01^*^ |  |  | 0.62 | 0.25 | 0.02^*^ |
| **Small** | 10 |  |  |  |  | 14 |  |  |  |
| Intercept |  | 110.55 | 1.60 | <0.01^**^ |  |  | 113.16 | 1.65 | <0.01^*^ |
| Baseline slope |  | -0.55 | 0.04 | <0.01^**^ |  |  | -0.59 | 0.04 | <0.01^*^ |
| Level change after intervention |  | 11.12 | 4.40 | 0.01^*^ |  |  | 10.53 | 5.59 | 0.06 |
| Slope change after intervention |  | 0.12 | 0.56 | 0.83 |  |  | 0.45 | 1.06 | 0.67 |
| **Medium** | 3 |  |  |  |  | 7 |  |  |  |
| Intercept |  | 174.80 | 1.75 | <0.01^*^ |  |  | 170.69 | 1.93 | <0.01^*^ |
| Baseline slope |  | -0.61 | 0.04 | <0.01^*^ |  |  | -0.55 | 0.05 | <0.01^*^ |
| Level change after intervention |  | 1.37 | 4.82 | 0.78 |  |  | -1.04 | 6.56 | 0.87 |
| Slope change after intervention |  | 0.19 | 0.61 | 0.76 |  |  | -0.64 | 1.24 | 0.61 |
| **Large** | 9 |  |  |  |  | - |  |  |  |
| Intercept |  | 438.99 | 4.07 | <0.01^*^ |  |  | - | - | - |
| Baseline slope |  | -1.98 | 0.10 | <0.01^*^ |  |  | - | - | - |
| Level change after intervention |  | 22.33 | 11.17 | 0.049^*^ |  |  | - | - | - |
| Slope change after intervention |  | 1.30 | 1.42 | 0.36 |  |  | - | - | - |

* P-value < 0.05

Table S3. Complete parameters of models, without autocorrelation adjustment, evaluating the impact of further MCAM policy intervention on the monthly average number of daily participants comparing the periods of pre-MCAM (i.e., PMES only, 2019/01~2019/08) vs. post-MCAM (i.e., PMES+MCAM, 2019/09~2019/12).

| Parameters in the model | PMES + add-on MCAM later | | | |
| --- | --- | --- | --- | --- |
|  | n | β | S.E. | P‐value |
| **Overall** | 34 |  |  |  |
| Intercept |  | 73.86 | 0.54 | <0.01^*^ |
| Baseline slope |  | -0.16 | 0.11 | 0.16 |
| Level change after intervention |  | -1.50 | 0.95 | 0.15 |
| Slope change after intervention |  | 0.28 | 0.33 | 0.41 |
| **Tiny** | 13 |  |  |  |
| Intercept |  | 35.19 | 0.53 | <0.01^*^ |
| Baseline slope |  | 0.38 | 0.10 | 0.01^*^ |
| Level change after intervention |  | 0.29 | 0.94 | 0.76 |
| Slope change after intervention |  | -0.62 | 0.32 | 0.09 |
| **Small** | 14 |  |  |  |
| Intercept |  | 81.50 | 0.87 | <0.01^*^ |
| Baseline slope |  | -0.15 | 0.17 | 0.42 |
| Level change after intervention |  | -3.11 | 1.55 | 0.08 |
| Slope change after intervention |  | 0.14 | 0.53 | 0.79 |
| **Medium** | 7 |  |  |  |
| Intercept |  | 130.39 | 0.58 | <0.01^*^ |
| Baseline slope |  | -1.20 | 0.11 | <0.01^*^ |
| Level change after intervention |  | -1.60 | 1.03 | 0.16 |
| Slope change after intervention |  | 2.23 | 0.35 | <0.01^*^ |

* P-value < 0.05
